# Non-pharmacological Treatment of Atrial Fibrillation†

**Published:** 2002-01-01

**Authors:** Ole-Gunnar Anfinsen

**Affiliations:** Department of Cardiology, Rikshospitalet University Hospital, N-0027 Oslo, Norway

## Abstract

In selected patients with atrial fibrillation and severe symptoms, non-pharmacological treatment may be an alternative or supplement to drug therapy. Atrioventricular nodal radiofrequency ablation (requires pacemaker implantation), or atrial pacing for sick sinus syndrome, are established treatment modalities. All other non-pharmacological therapies for atrial fibrillation are still experimental. After the Maze operation, atrial depolarization has to follow one specific path determined by surgical scars in the myocardium. This prevents new episodes of atrial fibrillation, but at a cost of perioperative morbidity and mortality. Catheter-based "Maze-like" radiofrequency ablation is technically difficult, and thrombo-embolic complications may occur. Paroxysmal atrial fibrillation sometimes is initiated by spontaneous depolarizations in a pulmonary vein inlet. Radio frequency ablation against such focal activity has been reported with high therapeutic success, but the results await confirmation from several centres. For ventricular rate control, most electrophysiologists presently prefer ablation to induce a complete atrioventricular conduction block (with pacemaker) rather than trying to modify conduction by incomplete block. Atrial or dual chamber pacing may prevent atrial fibrillation induced by bradycardia. It remains to confirm that biatrial or multisite right atrial pacing prevents atrial fibrillation more efficiently than ordinary right atrial pacing. An atrial defibrillator is able to diagnose and convert atrial fibrillation. The equipment is expensive, and therapy without sedation may be unpleasant beyond tolerability.

Atrial fibrillation is the most frequent sustained arrhythmia of clinical significance. The prevalence of chronic atrial fibrillation is about 0,5-1% [1]. It is rarely seen before the age of 50. However, Framingham data show a marked increase of atrial fibrillation by increasing age, approaching 10% in octogenarians (Table 1) [2].

Atrial fibrillation may be paroxysmal or chronic. The chronic form may develop after several reversible episodes, or may occur primarily. An episode of atrial fibrillation is defined as chronic if, somewhat arbitrarily, it is still present after seven days [3]. An attack that has not terminated within 48 hours is termed persistent atrial fibrillation. Pharmacological or electrical conversion is then usually necessary to re-establish sinus rhythm [3].

Atrial fibrillation may occur due to reversible factors such as alcohol ingestion, hyperthyroidism, or pulmonary embolism. In 70-80% of patients, atrial fibrillation is associated with organic heart disorders exemplified by coronary heart disease, hypertension with left ventricular hypertrophy, valve disease, or congenital heart defects. The number of patients with primary atrial fibrillation (lone fibrillation) decreases as more sophisticated diagnostic procedures are performed to expose underlying disease. Similarly, lone fibrillation is rare in elderly because subclinical heart disease proceeds and become manifest [3].

## Symptoms and clinical findings

Atrial fibrillation causes irregular and often rapid heart rate leading to palpitations, dyspnoea and asthenia. Hemodynamic function is impaired both at rest and during physical activity. The risk of cerebrovascular insults increases [2]. Thromboembolic complications are most frequently seen immediately after atrial fibrillation has started, during the first year of chronic atrial fibrillation, or early after conversion to sinus rhythm [3]. Several surveys show that cardiac as well as total mortality in patients with atrial fibrillation is doubled compared to sex and age-adjusted groups in sinus rhythm [4]. A major part of this increased risk is, however, attributable to underlying structural heart disease. Lone atrial fibrillation confers little risk, at least in persons below 60 years of age [5].

## Electrophysiologic substrate

The pathophysiological mechanism of paroxysmal and chronic atrial fibrillation is only partly understood. However, there is general agreement that the following factors contribute: Shortened refractory period, regional variations of refractoriness (dispersion of repolarization) and conduction velocity, and increased ectopic activity.

Gordon Moe described the substrate of atrial fibrillation as a continuous activation of the atrial myocardium by several reentry circles that are not anatomically fixed, but spread and mingle in a seemingly chaotic pattern [6]. The theoretical length of each reentry circle ("wavelength" = conduction velocity x refractory period) gives an expression of the minimal circumference each depolarization must travel to avoid that the electrical impulse reaches its origin before this is again excitable. When refractoriness is short and conduction velocity is slow, wavelength is also short. Thus, several reentry circles may exist simultaneously in the atria. Five or six reentry circles are associated with stable atrial fibrillation, while a situation with fewer reentry circles either converts to sinus rhythm, or degenerates into more reentry circles [7].

During the first hours to days of an attack of atrial fibrillation, electrophysiologic remodelling with gradual shortening of the refractory period has been found [8] . This implies stabilisation of the atrial fibrillation. Allessie and co-workers thus formed the thesis "Atrial fibrillation begets atrial fibrillation" [8] 

An atrial premature or escape beat may induce atrial fibrillation due to a "P-on-T-phenomenon". Some patients show frequent monomorphic extrasystoles, either singly or in long series. The origin may be found in the pulmonary vein inlets at a variable distance from the left atrium [9]. It is conceivable that electrical impulses from this focus (mother rotor) may induce chaotic activation of the atria due to interaction with anatomical and/or functional barriers that leads to fragmentation of the depolarisation front (daughter wavelets) [10]. It is not yet known how many patients really suffer from such focal atrial fibrillation. Small studies indicate 20% of primary atrial fibrillation [11], others suggest 30-40% of all paroxysmal atrial fibrillation .

## Pharmacological therapy

Most patients with atrial fibrillation are still treated pharmacologically. Antiarrhythmic therapy may serve one of three intentions: To convert atrial fibrillation into sinus rhythm, to prevent recurrence after cardioversion, or to achieve rate control in patients with permanent atrial fibrillation. Additionally, anticoagulation or antithrombotic therapy is indicated in several patients to prevent thromboembolic complications [12,13].

Paroxysmal atrial fibrillation frequently converts spontaneously if time is allowed to pass. In a study comparing different antiarrhythmic therapies, 76% of patients in the placebo group were in sinus rhythm within 48 hours [14]. Pharmacological conversion of atrial fibrillation may be achieved by flecainide (conversion rate for intravenous treatment: 59-93% [15] ). It remains to be seen which role the new class III-agents (dofetilide, ibutilide) will get. Sotalol has a limited conversion rate, but may be used similar to flecainide to prevent recurrence [16].

Kinidine has a well documented effect both concerning cardioversion and prophylaxis, but its use has been limited by side effects (diarrhoea and proarrhythmia). A classical meta-analysis showed that while only 25% of placebo treated patients were in sinus rhythm one year after electroconversion of atrial fibrillation, kinidine increased this fraction to 50% [17]. Newer drugs have not shown superior antiarrhythmic effect, but they are better tolerated. It is difficult to compare the prophylactic effect of different drugs between different studies, because inclusion criteria and duration of the arrhythmia may differ. Somewhat simplified, only 30-60% of patients will still be in sinus rhythm one year after a successful cardioversion, despite the use of drugs like flecainide, sotalol or amiodarone. Amiodarone both in studies and in daily clinic mostly has been reserved for resistant cases where the other drugs have failed.

Among patients with permanent atrial fibrillation, the therapeutic goal may be limited to rate control. Digoxin, b-blockers, verapamil or diltiazem are frequently prescribed. Digoxin may be combined with one of the others.

## Non-pharmacological therapy

The interest for non-pharmacological therapy has emerged due to the limited antiarrhythmic and frequent proarrhythmic effects of drugs. Thus, reasons for choosing non-pharmacological therapy may be paroxysmal atrial fibrillation with very frequent attacks and severe symptoms, chronic atrial fibrillation without adequate rate control leaving the patient at risk of developing tachycardiomyopathy, or patients who experience intolerable side effects of otherwise effective drug therapy. Additionally, in some patients with severe diastolic dysfunction due to for example left ventricular hypertrophy, the hemodynamic function may be grossly impaired if the driving force of the atrial systole disappears.

Non-pharmacological therapy includes several different treatment modalities ( Table 2 ). With the exceptions of radiofrequency ablation of the atrioventricular node (with pacemaker implantation), and atrial pacing in sick-sinus syndrome, all non-pharmacological therapy of atrial fibrillation is still experimental.

## Surgical treatment

Giraudon and co-workers in 1985 described the "corridor procedure", in which the right and left atrial free walls were isolated from the interatrial septum [18]. Rate control was achieved because the ventricles were controlled by the sinus node, but atrial myocardium continued to fibrillate. The risk of thromboembolic episodes therefore was unchanged. Today, this method has only historical interest.

**Figure 1:**
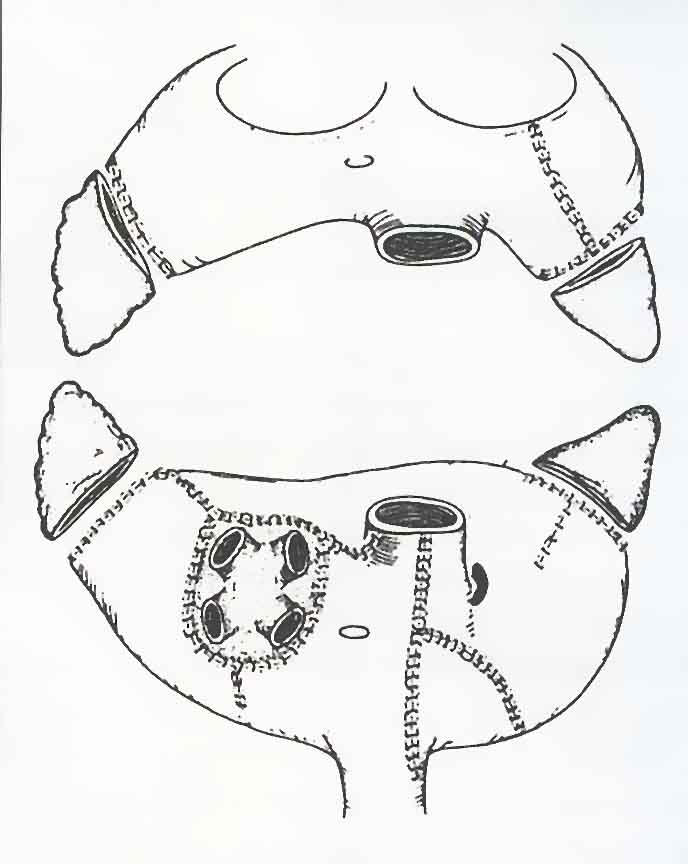
Schematic drawing of the right and left atria viewed from behind (below) or in front (above), with an indication of the surgical incisions that are created during Maze III operation. Compared to the original Maze procedure, the incisions in the right atrium are slightly modified to avoid damage to the blood supply of the sinus node [42]

The Maze procedure is performed by isolating the atrial appendages and cutting the atrial walls in a specific pattern (Figure 1 ) [19]. Thereby, the depolarization wave front is forced to follow one specific path from the sinus node to the atrioventricular node, and thus the atrial contraction will be organised. Reentry will not be possible, because the area of remaining continuous atrial myocardium is too small compared to the "wavelength" (see above) in atrial fibrillation [20] .  After the first publication, the pattern of incisions in the right atrium has been slightly modified to avoid injury to the sinus node artery leading to sinus node dysfunction postoperatively (Maze II and III). Cox found that 98% of his patients achieved atrioventricular synchronicity and mechanical atrial contraction, and that 60% showed acceptable chronotropic function of the sinus node. After Maze III surgery in patients where the sinus node function was documented to be normal preoperatively, less than 1% needed a pacemaker [21]. However, other authors have found that particularly the left atrial function will remain somewhat reduced compared to control patients. Larger patient cohorts and longer observation periods are needed to estimate any effect on thromboembolic episodes.

A Maze operation implies major heart surgery with an expected perioperative mortality of 1-2%. If the operation is performed in connection with valve surgery, one must expect about 70 minutes prolongation of the time spent on heart-lung machine. To shorten this time, some surgeons prefer to use cryoablation or radiofrequency ablation by a handheld probe instead of surgical incisions. Postoperatively, fluid retention is frequently observed, probably due to the absence of atrial natriuretic peptide. Several centres prefer the Maze operation before radiofrequency ablation of the atrioventricular node.

## Radiofrequency ablation

By delivering high-frequency (radiofrequency) alternating current through ablation catheters, it is possible to create small lesions inside the heart exactly at the anatomical substrate of various arrhythmias. This method may be curative for patients with atrioventricular reentry (overt or concealed Wolff-Parkinson-White syndrome) or atrioventricular nodal reentry tachycardias. Today, more than 90% success rate may be achieved, with few complications [22].

Atrial fibrillation in patients with Wolf-Parkinson-White-syndrome may be dangerous, because an accessory pathway with short refractory period causes very fast ventricular rate (pre-eexcited atrial fibrillation). If this accessory pathway is ablated, not only the atrioventricular reentry disappears, but also the risk of new episodes of atrial fibrillation has been shown to decrease. Similarly, atrioventricular nodal tachycardia may in some patients cause atrial fibrillation, and this may be prevented by "slow-pathway" ablation.

Except from these examples of arrhythmia-induced atrial fibrillation, radiofrequency ablation of atrial fibrillation is still limited. Ablation of the AV node to induce complete block and leave the patient pacemaker-dependant, is a purely palliative treatment. Some electrophysiologists try to modify atrioventricular conduction without inducing complete block. Research has partly aimed at developing a catheter-based "Maze-like" procedure, partly at revealing the pathophysiological mechanism of atrial fibrillation and potentially perform focal ablation.

### Induction of complete atrioventricular block

Historically, direct current ablation of the bundle of His and pacemaker implantation was the first non-pharmacological alternative to drug treatment of atrial fibrillation [23]. The procedure was performed in general anaesthesia, and might be complicated by serious ventricular arrhythmias or cardiac rupture. Therefore, a large step forward was taken in 1987 with the use of radio  frequency current [24], which gives a more localized and homogeneous tissue necrosis. High frequency alternating current does not stimulate pain fibres or neuromuscular end plates, so treatment could be performed in local anaesthesia.

AV-node ablation makes the patient pacemaker-dependant. Another argument against this treatment is the observation that some patients died suddenly during the first months after complete block had been created, both with direct current as well as with radiofrequency ablation. It is still unknown whether underlying heart disease caused this, or if the sudden rate reduction predisposed to arrhythmia. Geelen and co-workers found an incidence of 6% ventricular fibrillation or sudden death among 100 patients in whom the pacemaker was programmed at a heart rate of 70 or less. However, among the next 135 patients in whom the pacemaker during 1-3 months did not allow heart rates below 90, no cases of sudden death were found [25].

By radiofrequency ablation of the atrioventricular node, excellent rate control is achieved. Tachycardiomyopathy may be reversed, and the quality of life and physical fitness generally improve. For patients with paroxysmal atrial fibrillation, a dual chamber pacemaker with mode switch is recommended.

Published experience from the Arrhythmia Centre in Oslo contained 33 patients with atrial arrhythmias, of whom 23 had atrial fibrillation [26]. Complete atrioventricular conduction block was induced in 30 patients, while in two patients a partial block with satisfactory ventricular rate was achieved. The authors claim that irreversible destruction of normal conductive tissue must be restricted to patients with severe symptoms in whom other treatment strategies were not successful. One should particularly be reluctant to induce AV-block in young patients, hoping that the future may bring less mutilating therapy [26].

### Modification of the atrioventricular conduction

The ventricular rate in atrial fibrillation may be reduced by radiofrequency ablation in the same area as atrioventricular nodal tachycardias are treated, i.e. in the area of the slow pathway. This can be achieved without inducing a complete conduction block making the patient pacemaker dependant [27]. The treatment has been effective also in patients without dual nodal pathways. A possible explanation has been that ablation shifts the balance between stimulating and inhibiting input to the AV node. 60-70% of the patients achieves satisfactory ventricular rate control both at rest and during physical activity [28]. However, a little risk of ending up with a complete heart block remains, so this treatment should not be chosen in patients for whom a pacemaker is completely unacceptable. Some patients continue to have much discomfort, possibly because the heart rate still is very irregular although not that fast. Therefore, many electrophysiologists prefer to induce complete AV-block and implant a pacemaker primarily.

### Catheter-based compartmentalisation of the atria

**Figure 2:**
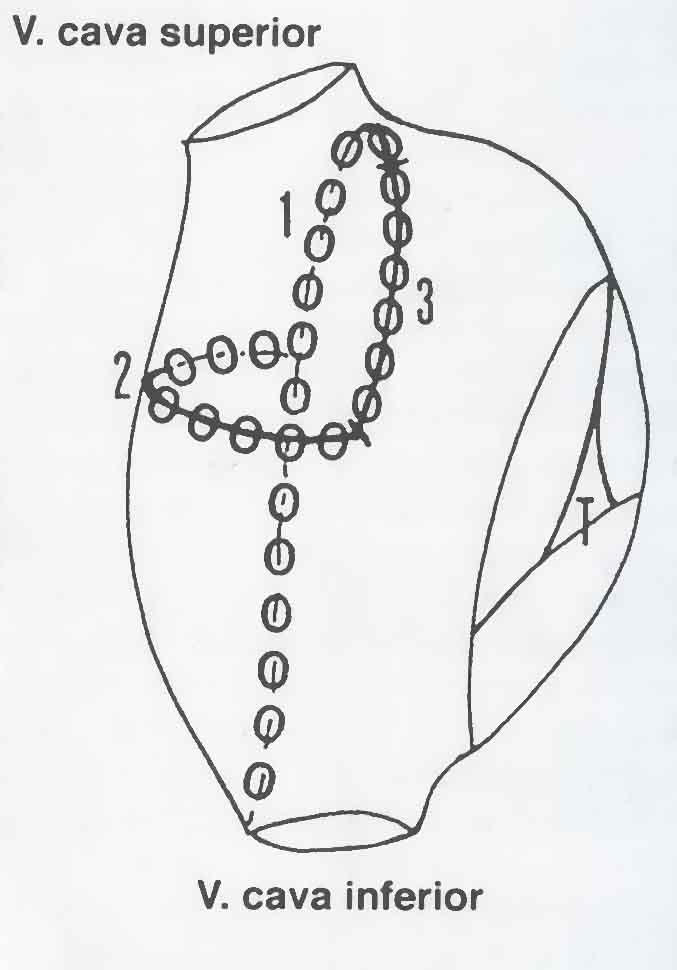
Schematic drawing of the right atrium and the three ablation lines posteriorly (1), horizontally on the lateral wall (2), and anteriorly (3) that were described in the first case published about curative treatment of atrial fibrillation by radiofrequency ablation [28]. This patient previously had been subjected to an inferior caval vein-tricuspid valve isthmus ablation due to atrial flutter.

Great efforts have been done to find a catheter-based technique with similar effect as the surgical Maze procedure. The concept again has been to reduce the size of confluent atrial myocardium so that multiple reentry circles are not allowed to establish. The first case description in this field was based on creating three lines of coagulation necrosis in the right atrium (Figure 2) [29]. However, subsequent experience with purely right-sided procedures has shown limited success.

Two major challenges have been faced: Where to place the linear lesions to prohibit atrial fibrillation [30], and how to secure that continuous transmural lesions have been created. Lesions in the left atrium that isolate the pulmonary vein inlets seem to be essential. However, large lesions in the left atrium imply a risk of thromboembolic complications. The pioneer JF Swartz stopped his efforts in ablating chronic atrial fibrillation after two of his 36 patients developed severe cerebrovascular insults. Biatrial ablation is time consuming. Initial experience showed total procedure duration approaching 12 hours, fluoroscopy time about 2 hours, and a frequent need for repeated sessions. Thus, one may ask whether the treatment is more dangerous than the illness. However, Swartz demonstrated that it is possible to achieve sinus rhythm with preserved biatrial mechanical function in 80% of the patients [30].

During subsequent Maze surgery in a patient in whom two non-successful attempts had been made to perform biatrial linear ablation, severe scarring and reduced function of both the right and left atria were found. The radiofrequency lesions were not transmural, and one pulmonary vein inlet was occluded by a thrombus [31].

The group of M Haissaguerre in Bordeaux (France) has particularly studied patients with paroxysmal atrial fibrillation. Using biatrial linear lesions in a pattern similar to that of Swartz', they report favourable results in 70% of the patients, with considerably less atrial fibrillation and less need for antiarrhythmic drugs [32].

## Radiofrequency ablation of focal atrial fibrillation

Atrial fibrillation may, at least in some patients, be induced by spontaneous depolarizations from a focus located in a pulmonary vein inlet (95%) or at various locations in the atrial myocard itself [9]. Atrial myocardial fibres have been found in the pulmonary veins as far as 5 cm from the inlet. The demonstration of focal atrial fibrillation opens for completely new therapeutic strategies, since limited ablation may be curative.

To diagnose supraventricular extrasystoles that induce atrial fibrillation, digital 12-channels Holter-recorders are needed. Radiofrequency ablation of a pulmonary vein inlet is performed by transseptal catheterization, and pulmonary venous angiography may be helpful. Specialized catheters have been designed to facilitate mapping and ablation of the pulmonary vein inlets. M Haissaguerre and co-workers have been pioneers also in this treatment [9]. Their experience indicate a total success rate without antiarrhythmic drugs around 70%, varying from 90% if a focus is located in one single pulmonary vein, to 20% if foci are located in all four pulmonary veins. Late stenoses of the pulmonary veins occurred in as much as 6% of patients, but among the first 150 patients, no specific treatment was needed. These results need to be confirmed at other centres.

New mapping techniques may be beneficial both for focal pulmonary vein ablation and for biatrial compartmentalisation procedures. Using electroanatomic mapping, a three-dimensional activation model of a cardiac chamber may be constructed by combining conventional activation mapping with determining the catheter position relative to an electromagnetic field ("Carto" system [33]). With non-contact mapping, virtual electrograms representing more than 3000 locations in a cardiac chamber may be determined simultaneously, allowing fast analysis of activation pattern ("Ensite" system [34]).

## Pacemaker therapy in atrial fibrillation

Patients with sick sinus syndrome may need a pacemaker to prevent severe bradycardia with syncope. Atrial fibrillation is a common part of the sick sinus syndrome (tachy-brady-arrhythmia). By atrial pacing, compared to ventricular pacing, total and cardiovascular mortality has been shown to decrease, and there is less thromboembolic complications as well as a reduced burden of atrial fibrillation [34,35].

Atrial pacing prevents long pauses and atrial escape beats, which may trigger atrial fibrillation. At the same time atrioventricular synchrony is possible. This is opposite to ventricular pacing, in which the atria are controlled by the sinus node, which may function poorly. Long pauses and an excessive dispersion of atrial repolarization may be expected. Atrial contraction against closed atrioventricular valves (pacemaker syndrome) may also cause atrial dilatation and contribute to atrial fibrillation.

This effect of atrial pacing to prevent atrial fibrillation is strictly prophylactic. Atrial pacing will not convert an attack of atrial fibrillation that has already started, because only a limited part of fibrillating atria will be captured. Thus, sinus pauses are the only indication of pacemaker therapy, while the effect on atrial fibrillation is secondary.

### Biatrial pacing

Daubert and co-workers tried to capture more of the atrial myocardium by stimulating both the right and the left atria simultaneously. Left atrial pacing was achieved by an electrode in the middle or distal part of the coronary sinus [36]. The SYNBIAPACE-study showed a trend towards a longer interval free of atrial fibrillation with biatrial compared to ordinary dual chamber pacing, but the difference did not reach statistical significance [37].

Saksena and co-workers performed multisite right atrial rather than biatrial pacing, by stimulating the high right atrium together with the coronary sinus ostium. Among 15 patients who prior to pacemaker implantation had an average of 1.5 episodes of atrial fibrillation per week, they found no such episodes during the first three months, when everybody was programmed to multisite right atrial pacing. During the next three months 12 patients were programmed to ordinary dual chamber pacing (three patients denied re-programming, and were excluded from this part of the study), and atrial fibrillation was observed in five patients [38]. Larger studies and longer observational periods are needed to confirm if pacing of the coronary sinus ostium really improves protection against atrial fibrillation.

## Atrial defibrillator

Cardioversion of atrial fibrillation by external synchronized direct current shocks has a high success rate. Internal defibrillation using catheters inside the heart is possible with lower current and even higher success rate. Due to the good results with implanted defibrillators for ventricular arrhythmia, one has also been interested in implantable defibrillators for paroxysmal atrial fibrillation [39].

A pure atrial defibrillator was available in 1998. Three electrodes were placed in the high right atrium, coronary sinus and right ventricle, respectively. Diagnosis of supraventricular arrhythmias was based on complex algorithms comparing heart rate and timing of electrical activity at the three electrodes. The shock was delivered between the two atrial electrodes.

A study of 51 patients followed during eight months showed that 96% of 227 episodes of atrial fibrillation were successfully converted. In 27% of the episodes, several shocks were necessary due to early relapses. No ventricular arrhythmia was elicited by the defibrillations [40]. This pure atrial defibrillator was, however, withdrawn from the market because the manufacturing company was bought. The new owners have signalled that they rather prefer a dual chamber defibrillator that is able to deliver low energy cardioversion in case of atrial fibrillation, but with the opportunity to deliver high energy shocks if ventricular arrhythmia should appear. Such a combined defibrillator is also available from another company.

The main argument against the atrial defibrillator, except that it is expensive, is that the patients feel the shock as painful. Some patients prefer hospitalisation and sedation prior to cardioversion, and then one may ask if anything really is gained by having the implanted atrial defibrillator. This treatment may, however, be useful for some patients with paroxysmal atrial fibrillation who do not have too frequent attacks (about one per month?), but with severe symptoms. It has been hypothesized that early defibrillation may prevent the electrophysiological remodelling and thereby inhibit the progression from paroxysmal to permanent atrial fibrillation [41].

## Conclusion

Non-pharmacological treatment of atrial fibrillation may be an alternative or supplement to drug therapy. Except for atrial pacing in patients with sick sinus syndrome and radiofrequency ablation of the atrioventricular node, all non-pharmacological treatments of atrial fibrillation are still considered as experimental. Some centres report excellent results with Maze surgery, while I doubt if catheter-based biatrial compartmentalisation will ever be a common treatment option. Focal ablation in the pulmonary veins is an exciting, new concept, but the results await confirmation from several centres. Atrial defibrillators may be a good option for selected patients who tolerate the shock. Future will show if it is possible to combine several of these therapeutic modalities, for example focal ablation and biatrial pacing, to increase the therapeutic success and reduce the risk of complications.

## Figures and Tables

**Table 1:**
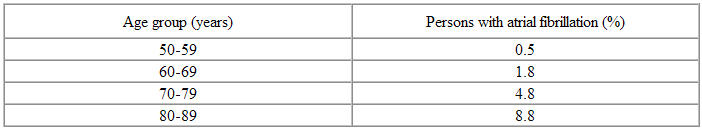
Number of cases with atrial fibrillation per 100 persons examined in the Framingham study [2]

**Table 2:**
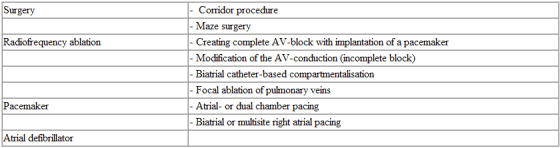
Various non-pharmacological treatment modalities for atrial fibrillation

**Table 3:**
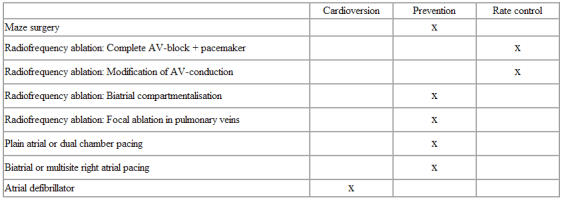
Therapeutic goals for non-pharmacological treatment of atrial fibrillation
